# Sjögren’s syndrome with nervous system injury combined with pulmonary and osseous cryptococcosis: a case report

**DOI:** 10.1186/s13256-021-02941-z

**Published:** 2021-06-25

**Authors:** Liping Xu, Xinwei Song, Yan Zhang, Na Lin, Ji-An Wang

**Affiliations:** Department of Rheumatology and Immunology, The First Affiliated Hospital of Zhejiang Chinese Medical University, Hangzhou, 310006 China

**Keywords:** Sjögren’s syndrome, Nerve damage, Pulmonary cryptococcosis, Osseous cryptococcosis

## Abstract

**Background:**

Sjögren’s syndrome is a common autoimmune disease that can involve the nervous system, but rarely both the central and peripheral. Long-term use of high-dose corticosteroids and immunosuppressants are the main risk factors for *Cryptococcus* infection in patients with Sjögren’s syndrome, of which pulmonary infection is the most common, while multiple bone infections are rare.

**Case presentation:**

A 46-year-old Chinese woman with a 2-year history of Sjögren’s syndrome presented to our hospital with numbness of limbs, shortness of breath, and weakness. Blood immunochemistry showed that antinuclear antibody (1:640), anti-Sjögren’s syndrome-A antibodies, and anti-centromere antibodies were strongly positive. Cranial magnetic resonance imaging revealed multiple demyelinating lesions in the white matter of bilateral cerebral hemispheres. Electromyography indicated serious peripheral nerve injury, especially in lower limbs. Computed tomography scan of lumbar vertebral displayed multiple high-density shadows, and the corresponding vertebrae on magnetic resonance imaging showed abnormal low signal intensity on T1 and T2 sequences. Positron emission tomography–computed tomography showed multiple lesions with high 18F-fluorodeoxyglucose uptake in lung and vertebral bodies. Both lung and bone biopsies suggested *Cryptococcus* infection, with the diagnosis of Sjögren’s syndrome with nervous system injury combined pulmonary and osseous cryptococcosis. She took a reduced dose of prednisone about 10 mg/day, terminated mycophenolate mofetil, and began to take immunoglobulin of 0.4 g/kg/day intravenously for 5 days, fluconazole (400 mg/day) for 6 months. Within 3 weeks, her chest radiography showed a marked improvement, and 3 months later, the pulmonary lesions disappeared on her computed tomography scan.

**Conclusions:**

This case exhibits an extremely rare condition of neural involvement in Sjögren’s syndrome combined with pulmonary and osseous cryptococcosis. This report also highlights the crucial role of detailed clinical examination, serologic markers, and biopsy in avoiding misdiagnosis. Currently, there is no guideline for this situation; in this case, we controlled the disease successfully with antifungal drugs and adequate gamma globulin, followed by an appropriate dose of corticosteroids.

## Background

Sjögren’s syndrome (SS) is a common autoimmune disease, can involve the nervous system, but rarely both the central and peripheral. In patients with connective tissue disease such as Sjögren’s syndrome with fungal diseases such as pneumocystis pneumonia (PCP), candidiasis, cryptococcosis, and penicilliosis, these fungal diseases are always induced by long-term therapy with immunosuppressant, but only a few case reports are describing multiple organ infection in patients. The authors report a case of Sjögren’s syndrome with nervous system injury combined with pulmonary and osseous cryptococcosis.

## Case presentation

A 46-year-old otherwise healthy Chinese female officer with a history of repeated limb numbness about 21 months was admitted to the Department of Rheumatology, the First Affiliated Hospital of Zhejiang Chinese Medical University, China on 4 November 2016. Without a history of tobacco and alcohol consumption, she suffered numbness of her right foot since February 2015 that soon spread to her limbs. Her electromyography examined in local hospital indicated peripheral nerve damage, her blood test showed antinuclear antibodies (ANA) 1:320 and anti-Sjögren’s syndrome-A (anti-SSA) antibodies positive, and her salivary gland Emission Computed Tomography (ECT) demonstrated low function, in addition, her labial gland biopsy revealed the number of infiltrating lymphocytes was more than 50 in 4 mm^2^ under the high-power field of vision. Thus, she was diagnosed with Sjögren’s syndrome and treated with methylprednisolone (MP) of 40 mg each day, hydroxychloroquine (HCQ) of 200 mg twice a day to suppress excessive immune inflammation, and immunoglobulin of 0.4g/kg/day for 5 days to modulate the immunity. Despite temporary relief, her limb numbness aggravated in March 2015. Then, she gained a prescription of mycophenolate mofetil (MMF) 1.0 g/day plus the same treatment as before, and her symptoms alleviated again. But she was hospitalized with a 1-month history of aggravated limb numbness, shortness of breath, and weakness in November 2016. There was no history of other illnesses. On examination, she had no fever (temperature 36.7 ℃), blood pressure (BP) 118/78 mmHg, heart rate (HR) 68 beats per minute, with decreased pulmonary sounds in the lower lobe of the left lung, and a few wet rales, as well as declined muscle strength about grade 4, with superficial sensory and positional hypoesthesia. The rest of her physical examination was negative.

**Fig. 1:**
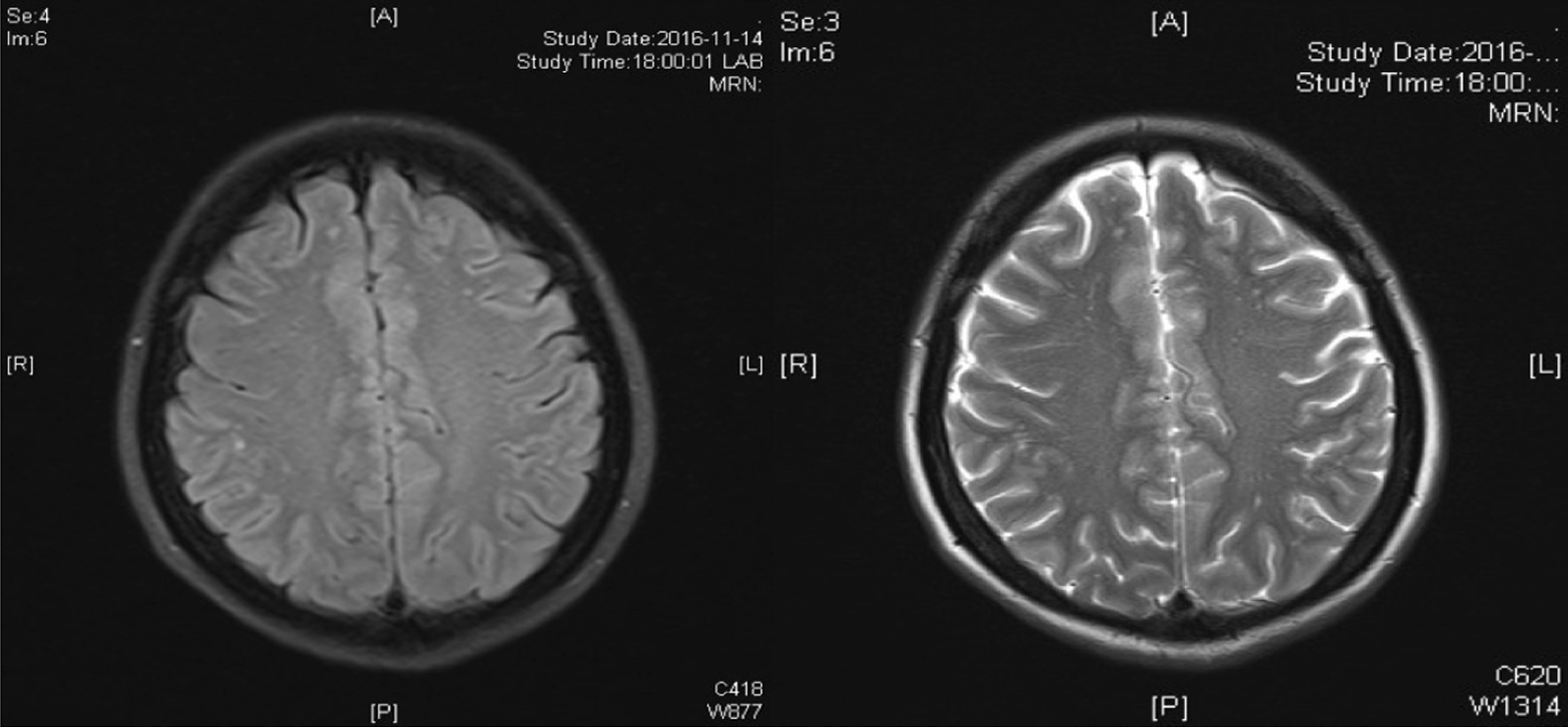
Cranial MRI revealing multiple demyelinating lesions in the white matter of both cerebral hemispheres

**Fig. 2:**
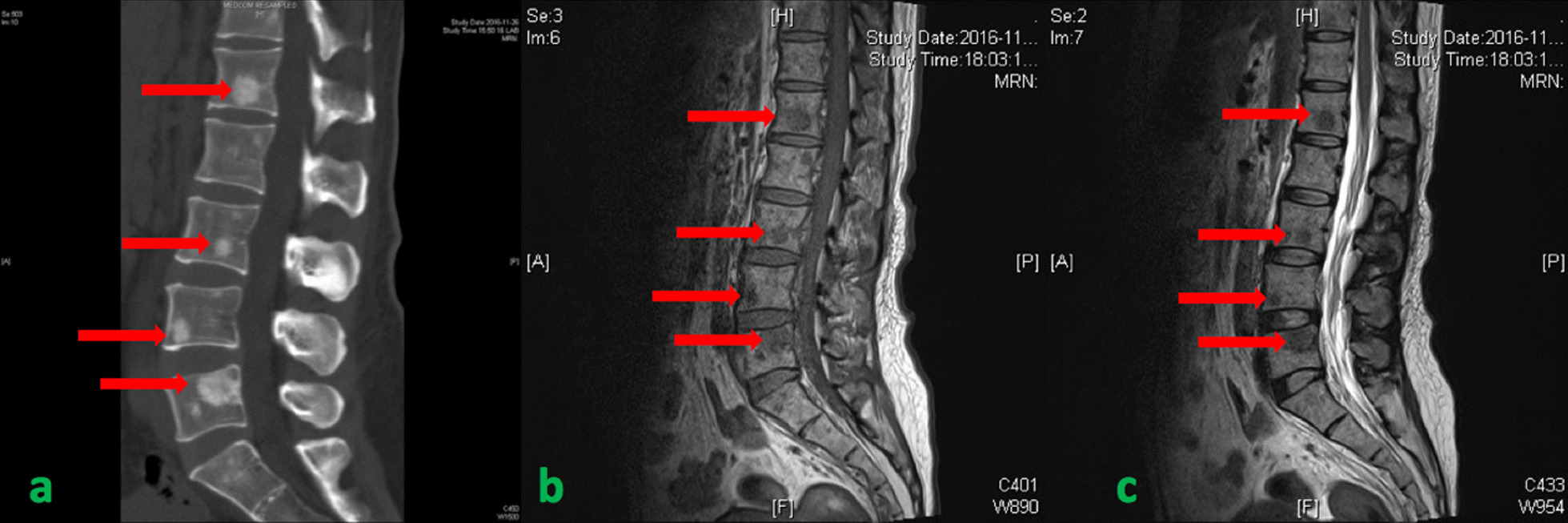
**a** CT scan showing multiple high-density lesions in lumbar vertebrae. **b**, **c** MRI demonstrating abnormal low signal in the corresponding vertebral bodies at T1 and T2 sequences. Red arrows point to the lesions

**Fig. 3:**
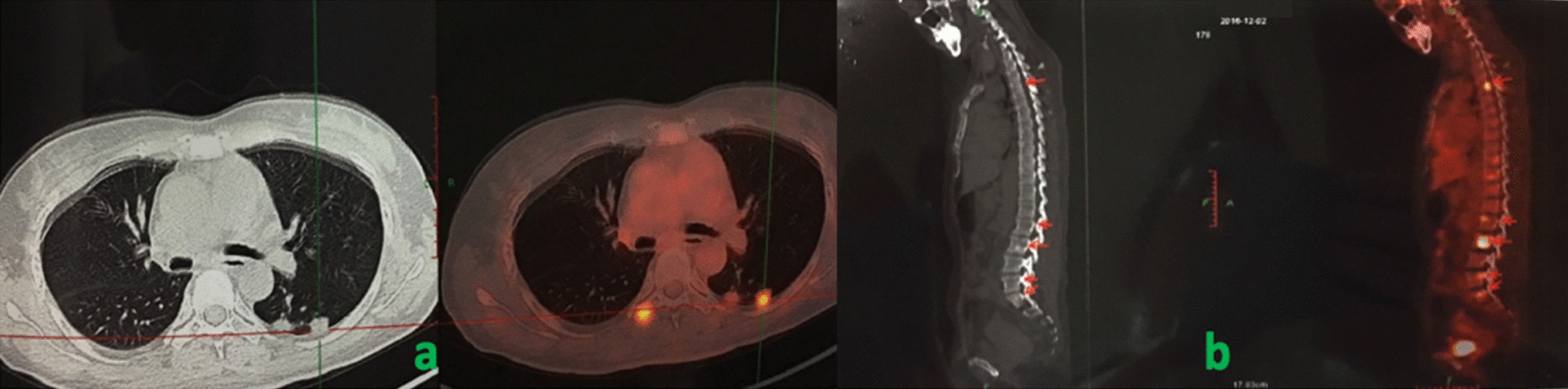
PET/CT showing multiple lesions with high 18F-FDG uptake in lung (**a**) and vertebral bodies (**b**)

**Fig. 4:**
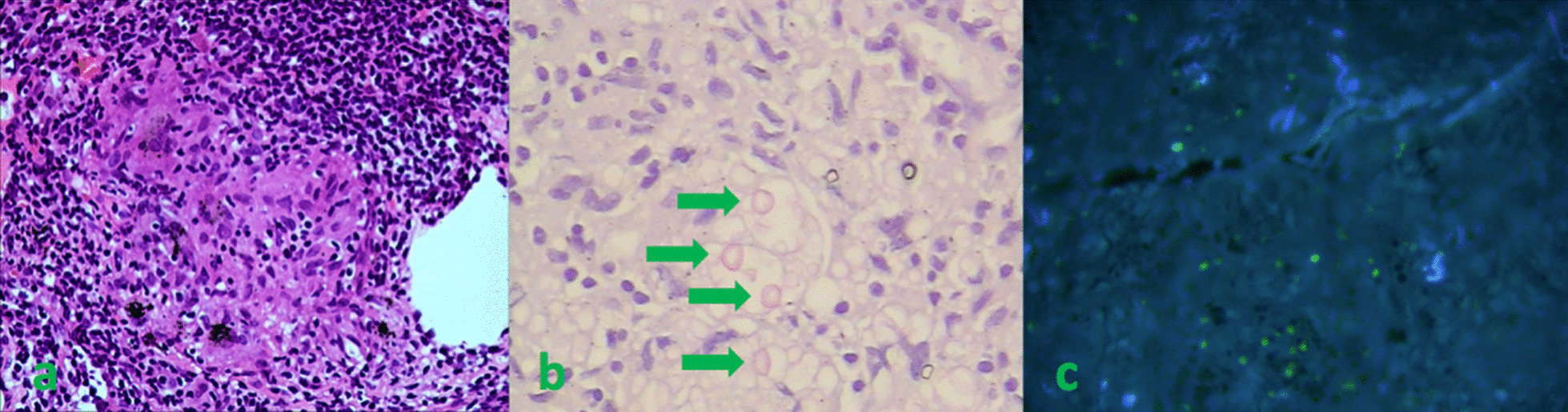
**a** Lung tissue biopsy showing granulomatous lesions [hematoxylin and eosin (HE), ×100]. **b** Lung tissue biopsy showing granulomatous lesions; green arrow points to *Cryptosporidium* [periodic acid–Schiff (PAS), ×400]. **c** Positive ink staining

**Fig. 5:**
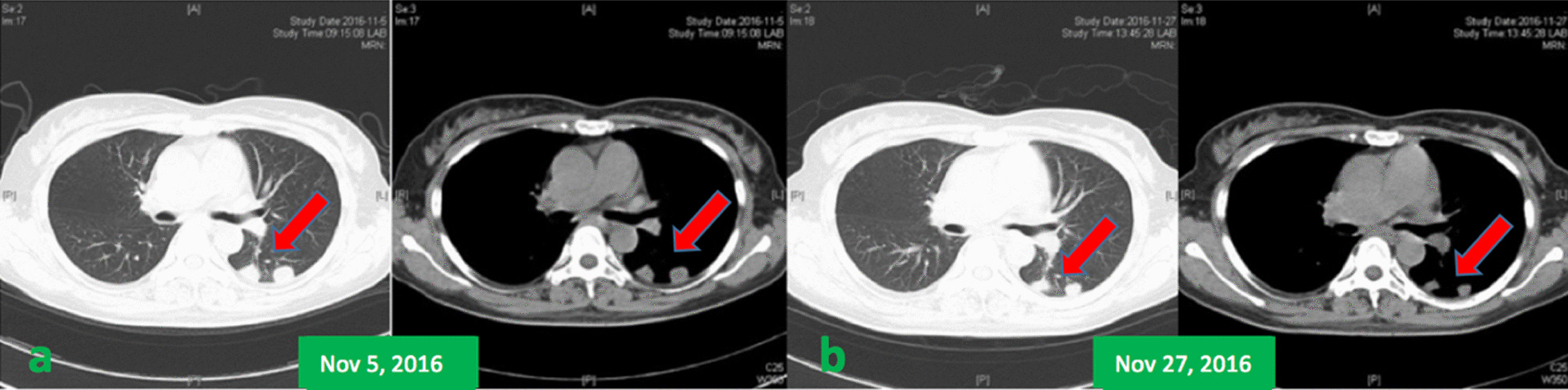
**a** CT scan showing multiple nodules in the left lung. **b** CT scan showing a reduction in lung lesions. Red arrows point to the lesions

According to the respiratory manifestation, a lung computed tomography (CT) scan was conducted, and the result showed multiple nodules in the lower lobe of the left lung and middle lobe of the right lung (Fig.5a), which was considered an infection; hence, we conducted possible pathogen tests, which revealed that serum cryptococcal capsular polysaccharide antigen was positive. Her sputum tests for bacteria, acid-fast bacilli, fungi, and serum G test (serum fungi-specific antigen detection), GM test (serum *Aspergillus*-specific antigen detection), T-SPOT (T-SPOT.TB test), human immunodeficiency virus (HIV), and rapid plasma reagin (RPR) test were negative. As for the evaluation of SS, serological tests showed that ANA was strongly positive at about 1:640, and anti-SSA and anti-centromere antibodies were positive, but the remaining immunologic tests were negative, including anti-neutrophilic cytoplasmic autoantibodies (ANCA), anti-glomerular basement membrane antibodies, anti-dsDNA, anti-Sm, and multiple tumor markers. Meanwhile, C-reactive protein and erythrocyte sedimentation rate (ESR) were normal. In addition, electromyography indicated serious damage in the limb-peripheral nerve, especially in lower limbs. Cranial magnetic resonance imaging (MRI) revealed multiple demyelinating lesions in the white matter of both cerebral hemispheres (Fig.1). CT scan displayed multiple high-density lesions in the lumbar vertebrae. MRI demonstrated an abnormally low signal in the corresponding vertebral bodies at T1 and T2 sequences (Fig. 2). Positron emission tomography–computed tomography (PET/CT) (Fig.3) showed multiple lesions with high 18F-fluorodeoxyglucose (18F-FDG) uptake in lung and vertebral bodies, and the radiologist believed that abnormal lesions were most likely to be cancer. Finally, her lung and bone tissue biopsies revealed granulomatous lesions with visible *Cryptosporidium* and positive ink staining (Fig.4) and negative acid-fast staining, which was consistent with *Cryptococcus* infection, and no *Cryptococcus neoformans* were found in the cerebrospinal fluid (CSF) .

She was eventually diagnosed with neurologic complications related to Sjögren’s syndrome combined with pulmonary and osseous cryptococcosis. The dose of prednisone was reduced to 10 mg/day, MMF was discontinued, and fluconazole (400 mg/day) was given for 6 months. Meanwhile, to modulate the immunity, immunoglobulin of 0.4 g/kg/day was injected intravenously for 5 days. Her CT scan showed that the lung lesions had reduced within 3 weeks (Fig.5b), and 3 months later, these lesions had disappeared. However, during this period, the symptoms of limb numbness worsened, indicating that SS was active. Although the infection was not fully controlled, she received therapy with prednisone (30 mg/day) since the 45th day after the treatment of fluconazole. Fortunately, her neurological symptoms and the infection were both controlled during the recent 1-year follow-up period.

## Discussion and conclusion

SS is a rare syndrome characterized by dry mouth and eyes, and 10–60% patients of suffer nervous system damage, with 2–25% in central nervous system [1]. Only a few cases of central and peripheral nervous system damage simultaneously in SS have been reported. Because the case of combined pulmonary and osseous cryptococcosis is rarer, we decided to report it.

Neurological involvement in Sjögren’s syndrome can be manifested as peripheral neuropathy and/or demyelinating encephalopathy, which is rare and highly malignant, and the pathology may be vasculitis [2], as in the patient we reported, whose cranial MRI displayed symmetrical demyelinating lesions according to the vascular distribution. Meanwhile, it has been reported that, in some patients with intracranial lesions, distribution of blood vessels is not consistent, showing multiple asymmetries, and it is speculated that the pathology may also be related to lymphocyte infiltration in the central nervous system [3].

The severity of the central nervous system lesions in SS is associated with anti-SSA antibody, but not with ANA or anti-SSB antibody [4], as in this case. Clinical manifestations of SS nervous system involvement are varied, repeatable, and multifocal, bearing a close resemblance to multiple sclerosis in symptoms, CSF, and imaging, but the age of onset, systemic symptoms, and serum antibodies are distinct.

The treatment for CNS involvement in SS, such as vasculitis, is recommended with 0.5–1 g glucocorticoid (GC) as the first-line therapy and cyclophosphamide as the second-line therapy [5]. However, in this case, the patient suffered pulmonary and osseous cryptococcosis at the same time; thus, we reduced the dose of prednisone, stopped the MMF, and gave an adequate dose of gamma-globulin for 5 days, and fluconazole for 6 months. In the later stage, we prescribed her a dose of prednisone 30 mg/day for SS treatment according to her aggravated neurological symptom related to SS.

In this case, the patient’s imaging examinations of the cervical and lumbar vertebral body indicated a wide range of lesions, and an 18F-FDG PET/CT revealed high metabolic FDG activation in lungs and bones, which was considered as metastatic tumors by radiologists. Data [6, 7] suggest that 18F-FDG PET scan can distinguish benign and malignant lesions by displaying the glucose metabolism of lesions. Despite high sensitivity and specificity, some inflammation and granulomatous lesions under PET can also show high 18F-FDG uptake, including cryptococcosis described in this case report, resulting in misdiagnosis.

*Cryptococcus neoformans* exists widely in nature, and it can infect lungs through the respiratory tract and spread to the brain, bone, and other tissues through blood circulation, especially in immunodeficient individuals [8], such as in this patient with a history of long-term glucocorticoid and immunosuppressant treatment. Despite multiple lesions in the lungs and bones, with PET/CT displaying potential metastatic tumors, her clinical symptoms did not support it; finally, she was diagnosed with cryptococcal infection by biopsy, and cured after antifungal therapy. It is worth mentioning that the literature review unveiled that it is not uncommon for patients with *Cryptococcus* infection to be misdiagnosed as having tumors.

We report the case of an extremely rare form of neural involvement in SS combined with pulmonary and osseous cryptococcosis. This case also highlights the crucial role of detailed clinical examination, serologic markers, imaging examinations, and biopsy to avoid misdiagnosis. Currently, there is no guideline for this situation, but a careful assessment of the extent and severity of system involvement would help us to produce the appropriate treatment strategy. In this case, the disease was successfully controlled with antifungal drugs and adequate gamma globulin, followed by an appropriate dose of corticosteroids.

## Data Availability

The datasets created during and/or analyzed during this case are available from the corresponding author on reasonable request.
